# Zinc finger transcription factor ZFP1 is associated with growth, conidiation, osmoregulation, and virulence in the *Polygonatum kingianum* pathogen *Fusarium oxysporum*

**DOI:** 10.1038/s41598-024-67040-7

**Published:** 2024-07-11

**Authors:** Jianyun Su, Jingyi Wang, Jingying Tang, Weimei Yu, Jiajia Liu, Xian Dong, Jiahong Dong, Xia Chai, Pengzhang Ji, Lei Zhang

**Affiliations:** 1 Institute of Medicinal Plant Cultivation, Academy of Southern Medicine, College of Chinese Materia Medica and Yunnan Key Laboratory of Southern Medicinal Utilization, Yunnan University of Chinese Medicine, Kunming, 650500 China; 2 Yunnan Normal University, Kunming, 650500 China

**Keywords:** Fungal biology, Fungal genetics, Fungal pathogenesis

## Abstract

Rhizome rot is a destructive soil-borne disease of *Polygonatum kingianum* and adversely affects the yield and sustenance of the plant. Understanding how the causal fungus *Fusarium oxysporum* infects *P. kingianum* may suggest effective control measures against rhizome rot. In germinating conidia of infectious *F. oxysporum*, expression of the zinc finger transcription factor gene *Zfp1*, consisting of two C_2_H_2_ motifs, was up-regulated. To characterize the critical role of ZFP1, we generated independent deletion mutants (*zfp1*) and complemented one mutant with a transgenic copy of ZFP1 (*zfp1* tZFP1). Mycelial growth and conidial production of *zfp1* were slower than those of wild type (*ZFP1*) and *zfp1* tZFP1. Additionally, a reduced inhibition of growth suggested *zfp1* was less sensitive to conditions promoting cell wall and osmotic stresses than *ZFP1* and *zfp1* tZFP1. Furthermore pathogenicity tests suggested a critical role for growth of *zfp1* in infected leaves and rhizomes of *P. kingianum*. Thus ZFP1 is important for mycelial growth, conidiation, osmoregulation, and pathogenicity in *P. kingianum*.

## Introduction

*Polygonatum kingianum* Coll. et Hemsl. is a native medicinal plant in Yunnan province, whose rhizome has medicinal and dietary concomitant functions^1,2^. However, rhizome rot seriously threatens the sustainable production of *P. kingianum*. The causal agents of rhizome rot in *P. kingianum* are *Fusarium oxysporum* and *F. solani,* and the former is more virulent^3^*. F. oxysporum* is a destructive soil-borne vascular fungal pathogen and the fifth largest plant pathogenic fungus globally that causes fusarium wilt, root rot, and necrosis, leading to severe yield losses in over 100 host plants^4–6^. However, there are few effective control methods against *F. oxysporum*, given the long-term survival of its chlamydospores in the soil and mycelia colonization in host xylem vessels^7^. Therefore, understanding the pathogenic mechanism of rhizome rot in *P. kingianum* and screening and identifying the *F. oxysporum* pathogenic genes can provide clues for exploring the scientific prevention and control measures against rhizome rot.

Transcription factors (TFs) regulate cell development, differentiation, and the cellular response to external perturbation by binding to a specific DNA site, or sites, where transcription activation or repression occurs through various mechanisms, including DNA–protein interactions, protein–protein interactions, and modification of the chromatin structure^8–10^. The zinc finger (ZF) protein is a TF with a ‘finger’ domain that regulates gene expression. It stabilizes a short polypeptide spatial configuration, folded into a finger-like structure by binding Zn^2^^+^. The ZF protein was first identified in *Xenopus* oocytes^11,12^, and is widely distributed in animals, plants and microorganisms^13^. It is divided into several subfamilies based on the numbers and positions of cysteine (Cys) and histidine (His) residues, including Cys_2_/His_2_-type (C_2_H_2_), C_2_HC, C_2_C_2_, C_2_HCC_2_C_2_, and C_2_C_2_C_2_C_2_^14^. More than 700 TFs have been predicted in *F. oxysporum* genome. However, only 26 TFs have been functionally analyzed, with the majority (15 TFs) belonging to the ZF protein family^15^. The 15 TFs include six Zn(II)_2_Cys_6_ ZFs, five C_2_H_2_ ZFs, two GATA ZFs, and two plant homeodomain (PHD)-containing ZFs^15^.

The *F. oxysporum* homolog of the TF *Ste12* which possesses a C_2_H_2_ domain, was up-regulated during the infection process, and was necessary for *F. oxysporum* virulence^16^. On the contrary, the pH signalling TF *PacC* with a C_2_H_2_ domain negatively regulated virulence, preventing the transcription of acid-expressed genes essential during *F. oxysporum* infection^17^. Zinc homeostasis regulator *ZafA* is also a C_2_H_2_ ZF. It was significantly up-regulated during the early stages of infection and was required for the full virulence of *F. oxysporum,* especially when zinc was limited^18^. However, *FolCzf1*, a C_2_H_2_ ZF in *F. oxysporum* f. sp. *lycopersici* (*Fol*), was required for growth, conidiation, conidia morphology, and pathogenicity in tomato^19^. Similarly, the *Con7-1* (a C_2_H_2_ ZF in *F. oxysporum*) deletion mutant exhibited defects in chitin synthase, hyphal branch, conidiation, and virulence^20^. Although these five C_2_H_2_ ZFs have been characterized in *F. oxysporum*, the function and regulation of most ZFs remain to be studied.

In this study, we identified ZF protein TF ZFP1 with two C_2_H_2_ domains, a homologue of *Fol* 4287 ZF protein MSN2/4^21^, whose function was still unknown. Therefore, this study aimed to investigate the roles of ZFP1 in the developmental processes and pathogenicity of the *F. oxysporum* in *P. kingianum*. The mutant *zfp1* and *zfp1* tZFP1 were generated by the target gene replacement technique. The gene *Zfp1* was up-regulated during *F. oxysporum* conidial germination*.* The inhibition rates, sensitivity under cell wall and osmotic targeted stresses, and virulence of *zfp1* were decreased compared to those of wild type *ZFP1* and *zfp1* tZFP1. The results elaborated the effects of ZFP1 on the growth, conidiation, stress response, and virulence of *F. oxysporum,* which detected an important gene that can be silenced using host-induced gene silencing for the prevention and control of the disease in the near future.

## Results

### Assessment of *Zfp1* expression pattern during conidial germination

**Figure 1:**
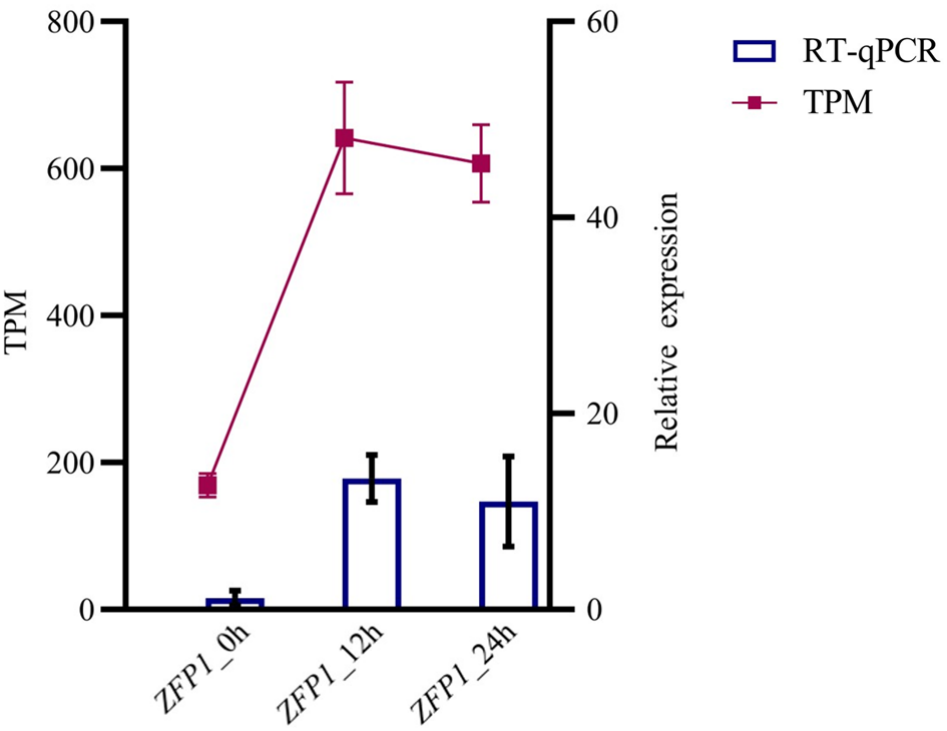
*Zfp1* expression pattern at 0, 12, and 24 h of conidia germination. Error bars represent standard deviation of the mean with three independent biological replicates. TPM means transcripts per kilobase of exon model per million mapped reads.

The transcriptional induction of *Zfp1* in germinating conidia was previously observed in published analysis of RNA sequencing^22^. To further assess the expression pattern of *Zfp1* during conidial germination, RNA was extracted at 0 h, 12 h, and 24 h and analyzed using RT-qPCR*.* The expressions of *Zfp1* in transcriptome data and RT-qPCR results were highly identical. The result indicated that expression of *Zfp1* was up-regulated in germinating conidia of infectious *F. oxysporum* (Fig. 1).

### ZFP1 is a C_2_H_2_-type ZF protein

**Figure 2:**
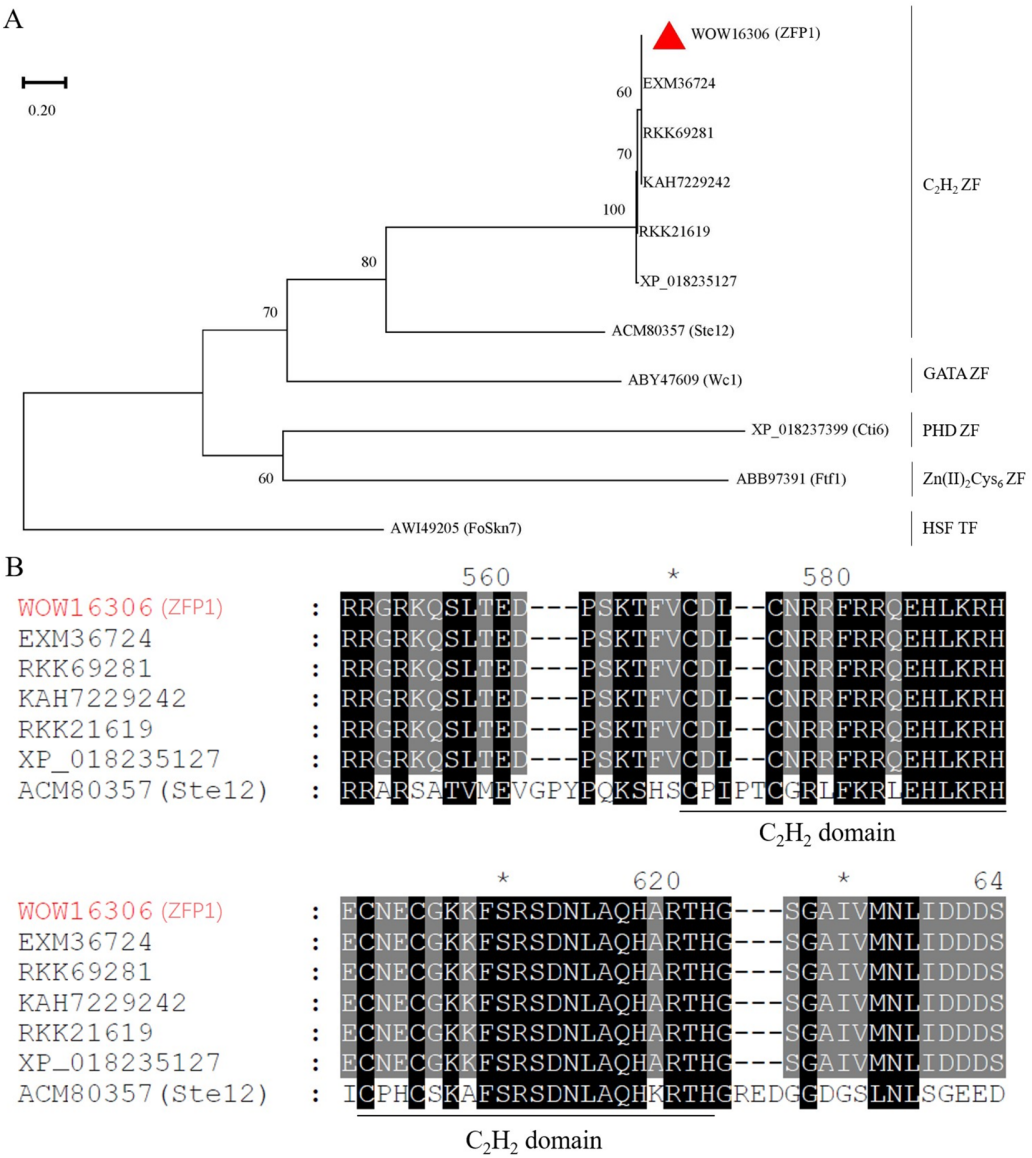
Phylogenetic relationships among *F. oxysporum* ZFs. (**A**) Phylogenetic tree constructed based on predicted amino acid sequences. Heat shock factor (HSF)-type TF was used as the outgroup. (**B**) Multiple alignment of predicted amino acid sequences of the C_2_H_2_ ZF region of *F. oxysporum*. Same amino acids were marked in black, and similar amino acids were shaded in grey. Black lines indicate the two C_2_H_2_ zinc-finger domains.

To analyze the structure of ZFP1, we performed BLASTn analysis in NCBI, and compared the deduced amino acid sequences of several ZF proteins by phylogenetic tree construction and multiple alignment. The gene length of *Zfp1* (GenBank accession no. OR715798), identified from genome sequence resource of *F. oxysporum* PkF01 (GenBank assembly accession no. JAMBQE000000000)^23^, was 1,836 bp; it contains one intron, and the length of coding sequence (CDS) was predicted to be 1,596 bp, encoding 531 amino acid residues. BLASTn analysis showed that *Zfp1* nucleotide sequence was 99% identity to the ZF gene MSN2/4 of *Fol* 4287 (FOXG_01955, GenBank accession no. XM_018379112). Additionally, a phylogenetic tree constructed based on the predicted amino acid sequences showed that ZFP1 (protein_id WOW16306) was classified into the C_2_H_2_-type subfamily, Ste12 (protein_id ACM80357)^16^ (Fig. 2A), and multiple alignment of predicted amino acid sequences showed that ZFP1 contained two C_2_H_2_ zinc finger domains (Fig. 2B), which making it a C_2_H_2_-type ZF protein.

### Generation of deletion mutant *zfp1* and its complementation by a transgenic copy of ZFP1

To study the critical roles of ZFP1, we generated three independent deletion mutants (*zfp1*-1, *zfp1*-2 and *zpf1*-3) by targeting *Zfp1* for gene replacement (as depicted in Fig. S1). Two DNA fragments were introduced into *F. oxysporum* using genetic transformation, one was sequence upstream of ZFP1 coding sequence fused with a partial hygromycin resistance gene, while the other was an overlapping partial hygromycin resistance gene fused to sequence downstream of ZFP1 coding sequence. Homologous recombination of the two DNA fragments and the ZFP1 genomic locus was expected to create a full-length hygromycin resistance gene that replaced coding sequence of ZFP1.

**Figure 3:**
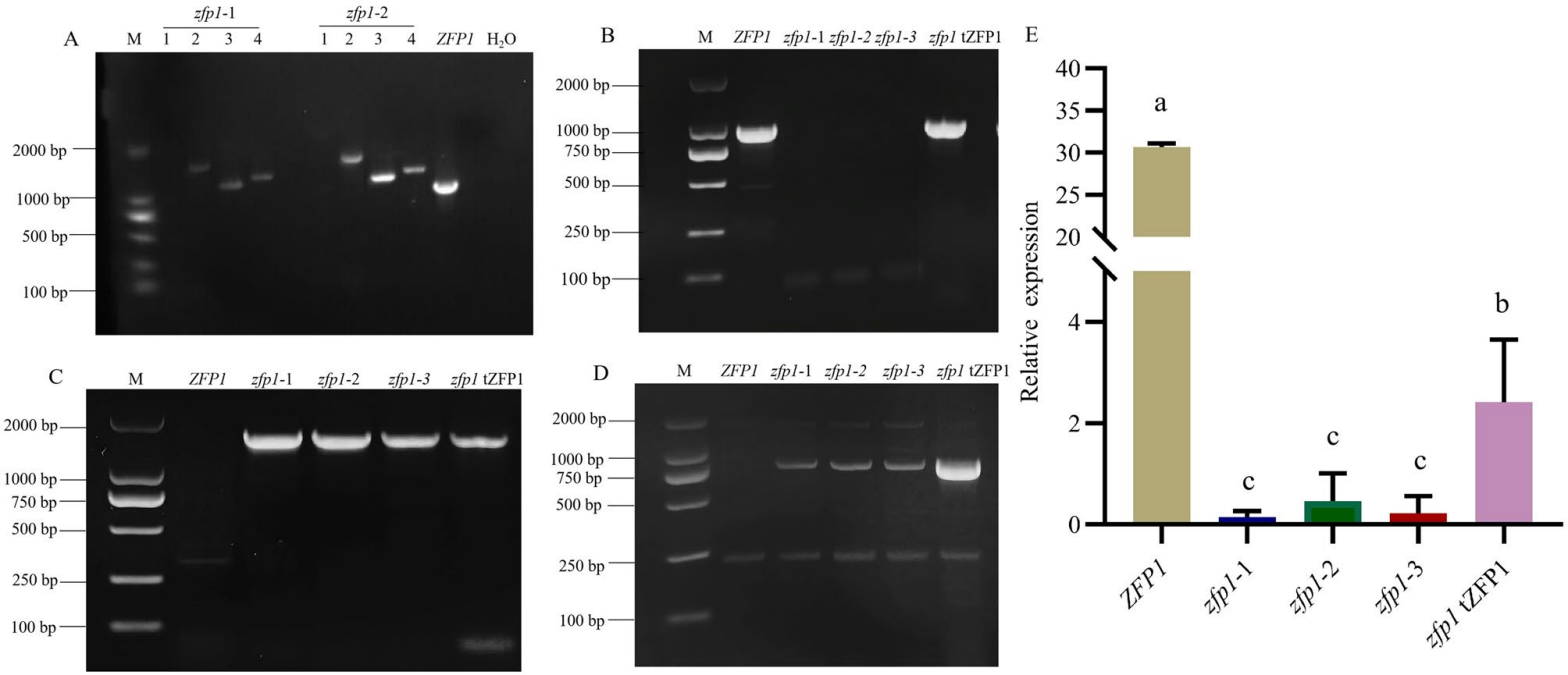
Verification of *zfp1* and *zfp1* tZFP1. (**A**): Verification of *Zfp1* by PCR. 1: Primer pair of *Zfp1*-IF/*Zfp1*-IR; 2: Hy-F/Yg-R; 3: *Zfp1*-UH-F*/Zfp1*-UH-R; 4: *Zfp1*-DY-F/*Zfp1*-DY-R. (**B**): *Zfp1* amplification with *Zfp1*-IF/*Zfp1*-IR. (**C**): Hygromycin B-resistant gene amplification with Hy-F/Yg-R. (**D**): Neomycin-resistant gene amplification with Neo-F/Neo-R. (**E**): Relative expression of *Zfp1* in *ZFP1*, *zfp1,* and *zfp1* tZFP1. Error bars represent standard deviation of the mean with three independent biological replicates. Different letters indicate significant differences at *P* < 0.05.

The correct gene replacement was verified using PCR, which showed that coding sequence of ZFP1 was successfully replaced in the hygromycin B-resistant transformants. Coding sequence of ZFP1 (942 bp) could be PCR-amplified from wild type *ZFP1* and not from *zfp1* mutants (Fig. 3A). Instead in the mutants three fragments of expected sizes (1,597 bp, 1,100 bp, 1,216 bp), corresponding to the full-length hygromycin resistance gene and fusions of hygromycin resistance gene to sequences upstream and downstream of ZFP1, were amplified respectively (Fig. 3A).

One mutant *zfp1* was complemented by integration of an ectopic copy of wild type *ZFP1*. Coding sequence of ZFP1 (942 bp) could be PCR-amplified from DNA of *ZFP1* and *zfp1* tZFP1 and not *zfp1* mutants (Fig. 3B). A hygromycin-resistance gene was PCR-amplified from the *zfp1* and *zfp1* tZFP1 transformant and neither from *ZFP1* (Fig. 3C). A neomycin-resistance gene, which was included in the transgenic construct and used to select for stable integration, was only PCR-amplified from the *zfp1* tZFP1 transformant and neither from *ZFP1* nor *zfp1* (Fig. 3D). Expression of *Zfp1* was significantly reduced in *zfp1* mutants and restored in *zfp1* tZFP1 (Fig. 3E). These results validated the loss of ZFP1 in *zfp1* mutants and transgenic complementation of *zfp1* in *zfp1* tZFP1.

### Deletion of *Zfp1* affects mycelial growth and conidial formation

**Figure 4:**
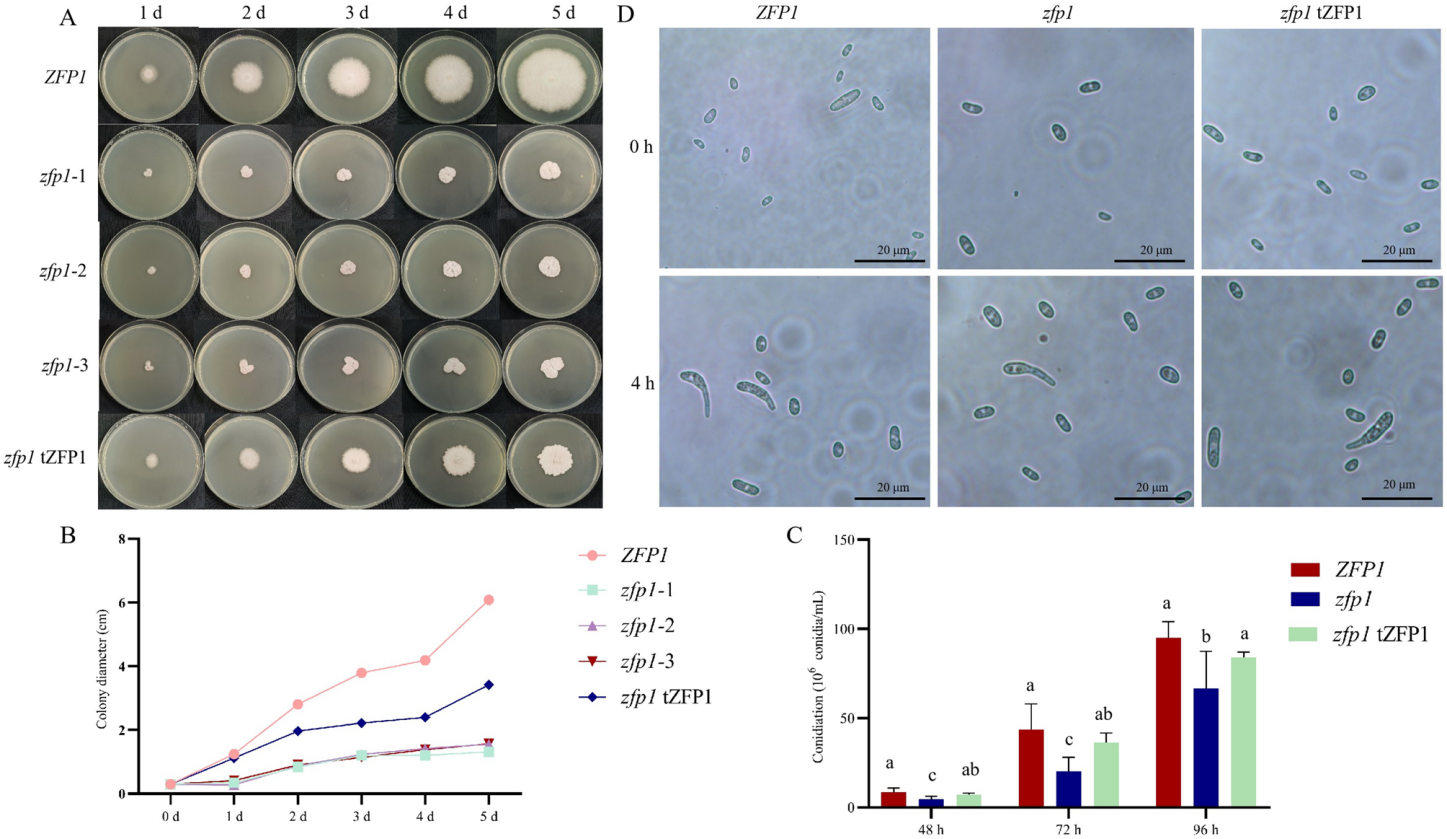
*ZFP1*, *zfp1*, and *zfp1* tZFP1 mycelial growth and conidiation. (**A**): Colony morphology on PDA; (**B**): Colony diameters on PDA; (**C**): Conidiation in PDB. (**D**): Conidial morphology. Error bars represent standard deviation of the mean with three independent biological replicates. Different letters indicate significant differences at *P* < 0.05.

To investigate the functions of *Zfp1* deletion in vegetative growth and asexual reproduction, we determined growth rates and conidiation of wild type *ZFP1*, *zfp1* and *zfp1* tZFP1. The *zfp1* colony formation and growth rates were significantly reduced compared to *ZFP1* and *zfp1* tZFP1 cultured on potato dextrose agar (PDA) for 5 days (Fig. 4A, B). Furthermore, a significantly reduced number of micro-conidia was produced in *zfp1* from 48 to 96 h post-inoculation on potato dextrose broth (PDB) (Fig. 4C). However, *zfp1* conidial morphology was not different compared to *ZFP1* and *zfp1* tZFP1 at 4 h incubation in PDB (Fig. 4D). These results indicated that deletion of *Zfp1* affected mycelial growth and conidial formation in *F. oxysporum*.

### Deletion of *Zfp1* reduces virulence

**Figure 5:**
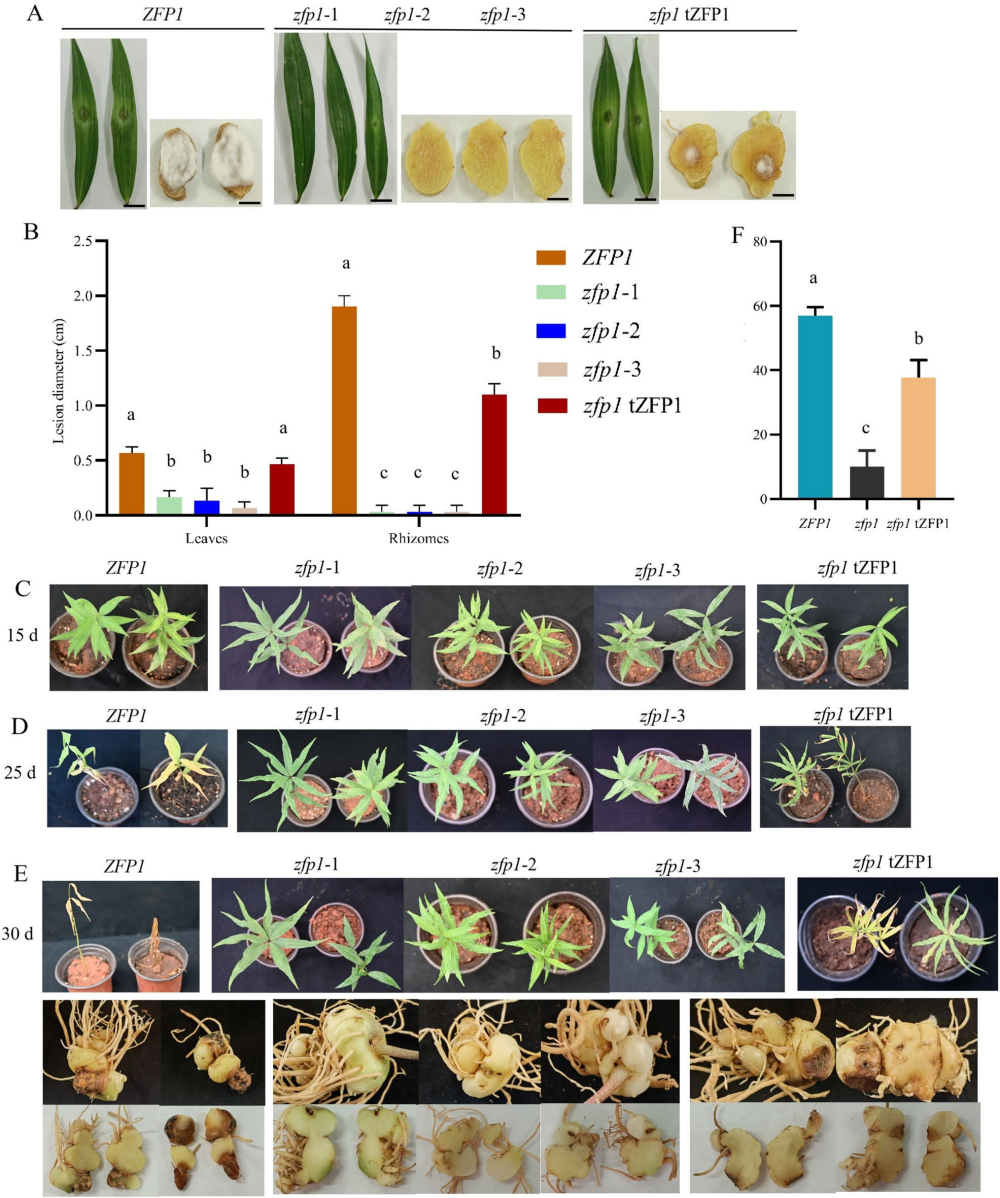
*ZFP1*, *zfp1*, and *zfp1* tZFP1 virulence in *F. oxysporum*. (**A**): Symptoms on detached leaves and rhizomes inoculated with *ZFP1*, *zfp1*, and *zfp1* tZFP1 for 5 days. (**B**): Lesion diameters of leaves and rhizomes inoculated with *ZFP1*, *zfp1*, and *zfp1* tZFP1 after 5 days of inoculation. (**C**): Symptoms of plants inoculated with *ZFP1*, *zfp1*, and *zfp1* tZFP1 after 15 days. (**D**): Symptoms of plants inoculated with *ZFP1*, *zfp1*, and *zfp1* tZFP1 after 25 days. (**E**): Symptoms of plants inoculated with *ZFP1*, *zfp1*, and *zfp1* tZFP1 after 30 days. (**F**): Disease index at 30 days post-inoculation. Scale bars: 10 mm. Error bars represent standard deviation of the mean with three independent biological replicates. Different letters indicate significant differences at *P* < 0.05.

To investigate the effects of *Zfp1* deletion on virulence, we inoculated detached leaves, detached rhizomes, and whole plants with conidial suspension of wild type *ZFP1*, *zfp1* and *zfp1* tZFP1. In the infection assays, the *zfp1* lesion diameters in the detached leaves and rhizomes were significantly decreased compared to *ZFP1* and *zfp1* tZFP1 (Fig. 5A, B). Withering of the leaves and rhizome rot were observed among the *P. kingianum* plants inoculated with *ZFP1* and *zfp1* tZFP1. However, no symptoms were observed in *zfp1* (Fig. 5C-E). Overall, the disease index of *ZFP1* and *zfp1* tZFP1 was significantly higher than that of *zfp1* (Fig. 5F)*.* These results demonstrated that *zfp1* strongly reduced the virulence of *F. oxysporum* on *P. kingianum.*

### ZFP1 contributes to *F. oxysporum* stress responses

**Figure 6:**
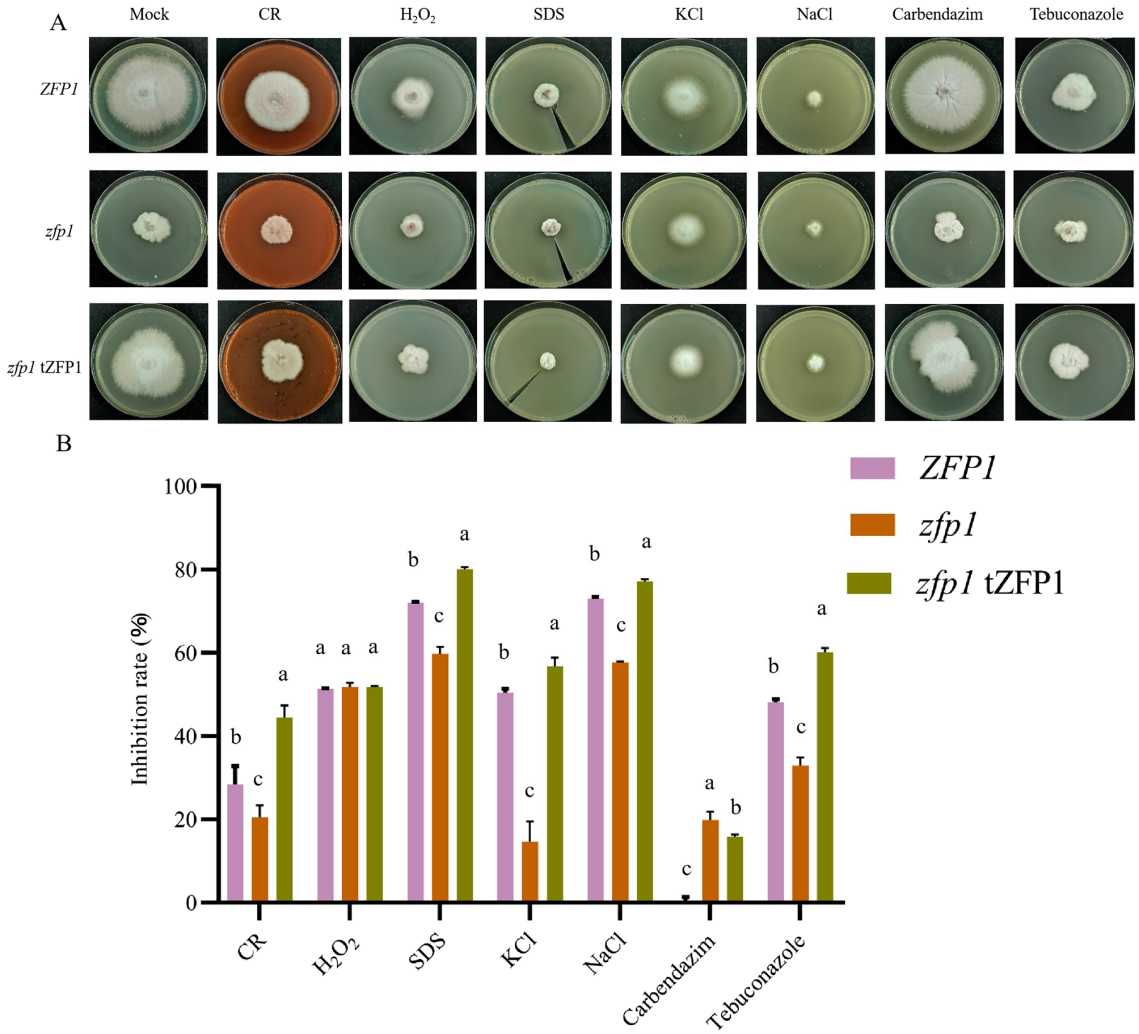
*ZFP1*, *zfp1*, and *zfp1* tZFP1 vegetative growth under different stressors. (**A**) *ZFP1*, *zfp1*, and *zfp1* tZFP1 colony morphology on PDA (mock) and PDA supplemented with 0.1% CR, 0.08% H_2_O_2_, 0.05% SDS, 1.8 M KCl, 1.8 M NaCl, 0.4 μg·mL^-1^ carbendazim, and 0.25 μg·mL^-1^ tebuconazole for 5 days. (**B**) The relative inhibition rates based on colony diameters at 5 days post-incubation. Error bars represent standard deviation of the mean with three independent biological replicates. Different letters indicate significant differences at *P* < 0.05.

To investigate the effects of ZFP1 on *F. oxysporum* adaptation to infection-related stresses, we compared the radial growth rates of wild type *ZFP1*, mutant *zfp1*, and *zfp1* tZFP1 on PDA under cell wall stresses (congo red, CR; sodium dodecyl sulfate, SDS), oxidative stress (H_2_O_2_), osmotic stresses (NaCl, KCl), tebuconazole and carbendazim stresses. The inhibition rates and sensitivity of *zfp1* under CR, SDS, NaCl, KCl, and tebuconazole stresses were decreased compared to *ZFP1* and *zfp1* tZFP1. However, *zfp1* had a lower tolerance to carbendazim compared to *ZFP1* and *zfp1* tZFP1. There was no significant difference in the inhibition rates of *ZFP1*, *zfp1,* and *zfp1* tZFP1 under H_2_O_2_, suggesting that ZFP1 had little effect on the sensitivity to oxidative stress (Fig. 6). These results suggested that ZFP1 was involved in regulating responses to cell wall integrity stresses and osmotic pressure in *F. oxysporum*.

## Discussion

Rhizome rot is a devastating soil-borne disease, seriously threatening the *P. kingianum* industry. *F. oxysporum* invades the roots, causing wounds, colonizes the vascular tissues, blocking water and nutrient transport*,* and may even lead to plant death^24^*.* Although over 700 TFs have been predicted in *F. oxysporum*, only 15 ZFs are associated with pathogenicity^15^. Among them is *Fow2*, a Zn(II)_2_Cys_6_-type transcription regulator, essential for root invasion and colonization but not for vegetative growth and conidiation in *F. oxysporum*^25^. The cutinase transcription factors *ctf1* and *ctf2,* containing the Zn_2_Cys_6_ DNA binding domain, also play important roles in the lipolytic system of *Fol*. Additionally, *ctf1* and *ctf2* deletion mutants severely reduce *F. oxysporum* virulence^26^. In common bean, *Fusarium* transcription factor 1 (*ftf1*) with a Zn(II)_2_Cys_6_ motif is only up-regulated during plant infection, where multiple *ftf1* copies increase virulence in *F. oxysporum* f. sp. *phaseol*^27^. Additionally, *EBR1*, belonging to the Zn_2_Cys_6_ family, regulates the expression of genes encoding metabolism and virulence. *EBR1* deletion impairs growth and reduces pathogenicity and biocontrol capacities in different *F. oxysporum* strains^28^. Furthermore, the global nitrogen regulator *FNR1*, with a single conserved GATA-type ZF domain, regulates the secondary nitrogen acquisition in plants. Notably, the disruption of *FNR1* mutants significantly delays *F. oxysporum* infection in tomato seedlings^29^. Besides, *Cti6*, which contains a PHD finger motif and simultaneously interacts with the transcriptional corepressor complex Cyc8-Tup1 and the co-activator SAGA (Spt-Ada-Gcn5-acetytransferase) complex, is required for full virulence in *F. oxysporum* on tomato^30^. In this study, we identified C_2_H_2_ ZF ZFP1 (protein_id WOW16306) was 88%, 57%, and 46% identity with *F. graminearum* GzC2H045 (protein_id XP_011326528)^31^, *Verticillium dahliae* VdMsn2 (protein_id XP_009648969)^32^, and *Magnaporthe oryzae* MoMSN2 (protein_id MGG 00,501)^33^, respectively. Disruption of these three genes all had defects on hyphal growth and virulence, which were similar to those of *zfp1.* However, the regulatory mechanism in pathogenicity needs to be further analyzed in the plant-pathogen interaction, which includes investigation the proteins interacting with ZFP1 by yeast two-hybrid technique, prediction the location of *Zfp1* by subcellular location, and detection the differences of fusarium acid content between wild type *ZFP1* and mutant *zfp1,* etc.

The host immune system first detects the pathogenic fungi conidia during the infection process. With *F. oxysporum*, conidial germination is the key step of infection^34^. Therefore, early conidial detection is crucial to inhibit fungal growth and alleviate the disease occurrence^35^. In this study, C_2_H_2_ ZF *Zfp1* was screened using the transcriptome data of the conidial germination process in *F. oxysporum*^22^. *Zfp1* was significantly up-regulated during conidial germination, suggesting that this gene might be related to *F. oxysporum* growth and pathogenicity. To verify this conjecture, *zfp1* and *zfp1* tZFP1 were constructed using split-marker homologous recombination, which revealed that ZFP1 regulated mycelial growth and conidial yield and thus the virulence of *F. oxysporum*. However, this did not affect the conidia morphology. Similarly, *BcTaf14*, TATA box-binding protein-associated factor 14 (Taf14) in *Botrytis cinerea* was associated with mycelial growth, conidiation, and conidial morphogenesis with no effect on conidial germination^36^. *snt2*, a PHD-containing ZF, was also essential in vegetative growth, conidial production, and host colonization by *F. oxysporum* f. sp. *melonis*^37^. However, the white-collar 1 photoreceptor *Wc1*, a GATA ZF, played roles in the hyphae development of *F. oxysporum*, but was dispensable for pathogenicity on tomato plants^38^.

When a pathogen invades the host, the host exhibits some defensive response, including a change in the internal environment or vascular system^39^. Similarly, pathogenic fungi always adopt a series of complex strategies for successful invasion of the host, including the plant defense mechanisms, host intracellular environment, and defeating adverse environmental changes^40,41^. In this study, *zfp1* increased resistance to membrane stressor (SDS), cell wall stressor (CR), extracellular osmotic (NaCl), and intracellular osmotic (KCl) stress compared to the *ZFP1*. However, no differences were identified in tolerance to oxidative (H_2_O_2_) stress between the mutants *zfp1* and the wild type *ZFP1*, implying that ZFP1 regulated osmotic pressure and cell wall integrity stresses. These results are similar to those of *Aoime2* (*Arthrobotrys oligospora* inducer of meiosis 2) deletion mutants which were unaffected by oxidative stressor H_2_O_2_ but highly sensitive to the osmotic stressor NaCl^42^. Similarly, *BcTaf14* deletion mutants increased NaCl and KCl sensitivity^36^. Neutral trehalase-encoding gene *NTH1* knockout mutant was also sensitive to H_2_O_2_ and SDS but not to CR, NaCl, and KCl^43^. On NaCl plates, there was almost no radial growth of *zfp1* and *ZFP1*. Interestingly, growth of *zfp1* and *ZFP1* appeared to be more comparable on plates with KCl, suggesting that KCl was suppressing the defect of *zfp1*.

In conclusion, the C_2_H_2_ ZF protein ZFP1 plays significant roles in mycelial growth, conidiation, stress response, and virulence in *F. oxysporum* of *P. kingianum.* However, how ZFP1 exerts these functions requires further study.

## Materials and methods

### Isolation of the fungal strain and the culture conditions

The wild-type *F. oxysporum* strain PkF01 isolated from the *P. kingianum* rhizome rot samples, was identified with nucleotide sequences of the elongation factor 1-alpha (GenBank accession no. MW149127) and the second largest subunit of nuclear DNA-directed RNA polymerase II (GenBank accession no. MW194100) by L. Zhang in our previous study^3^. For conidia production, mycelia were incubated in PDB at 28 °C with shaking at 180 revolutions per minute (rpm) for 3 days. Subsequently, the conidial suspension was adjusted to 1 × 10^6^ conidia·mL^-1^, and 30% glycerol was added before storing the suspension at − 80 °C^44^.

### Phylogenetic tree construction and protein sequence alignment

*Zfp1* nucleotide sequence was submitted to the National Center for Biotechnology Information (NCBI) GenBank database, and BLASTn analysis was performed in NCBI. To further investigate the function of ZFP1 in *F. oxysporum*, phylogenetic tree and multiple alignment of the C_2_H_2_ zinc-finger proteins of *F. oxysporum* were performed using the maximum likelihood method with 1,000 replications of bootstrap in MEGA 11^45^ and edited in GeneDoc^46^, respectively.

### Generation of *Zfp1* deletion mutants (*zfp1*)

The *Zfp1* gene in *ZFP1* was deleted using the split-marker recombination technology^47,48^. Firstly, 678 bp upstream (*Zfp1*-Up) and 835 bp downstream (*Zfp1*-Down) *Zfp1* fragments, and 800 bp upstream (Hy) and 1,112 bp downstream (Yg) hygromycin B-resistance cassette (HYG) fragments from the vector, pZD101-AmCyan^48^ were amplified using four sets of primer pairs *Zfp1*-UF/*Zfp1*-UR, *Zfp1*-DF/*Zfp1*-DR, Hy-F/Hy-R, and Yg-F/Yg-R, respectively. Secondly, *Zfp1*-Up and Hy were fused through PCR splicing by overlap extension^49^, using the *Zfp1*-UF/Hy-R primer pair and *Zfp1*-Up/Hy as the templates. Additionally, *Zfp1*-Down was fused with Yg using the Yg-F/*Zfp1*-DR primer pair and *Zfp1*-Down/Yg as the template (Fig. S1). Thirdly, the two fusion fragments were transformed into *ZFP1* protoplasts following the polyethylene glycol-mediated protoplast transformation technique^50^. Finally, the transformants were screened on PDA containing ampicillin (100 mg·L^-1^) and hygromycin B (400 mg·L^-1^).

### Complementation of *Zfp1* deletion mutant (*zfp1* tZFP1)

*Zfp1* was complemented with a 1,848 bp fragment containing the complete open reading frame of *Zfp1*. The fragment amplified with *Zfp1*-CF/*Zfp1-*CR primer pair was cloned in frame with the strong constitutive *Aspergillus nidulans gpdA* promoter contained in vector pDHtsk-GFP-G418 with a neomycin-resistant cassette. Next, the recombinant plasmid was transformed into *zfp1* protoplasts. Subsequently, the transformants were screened on PDA containing hygromycin B (400 mg·L^−1^) and neomycin (300 mg·L^−1^).

### Verification and quantification of gene expression

The deletion mutants were verified by PCR using primer pair *Zfp1*-IF/*Zfp1*-IF, *Zfp1*-UH-F/*Zfp1*-UH-R, and *Zfp1-*DY-F*/Zfp1-*DY-R. The complementary mutants were verified using *Zfp1*-IF/*Zfp1*-IR, Hy-F*/*Yg-R, and Neo-F/Neo-R primer pairs. RT-qPCR further validated *Zfp1* expression. RNA extraction, cDNA synthesis, and RT-qPCR were performed with TaKaRa MiniBEST Universal RNA Extraction Kit (TaKaRa, Code No. 9767), PrimeScript™ RT Master Mix (TaKaRa, Code No. RR036A), and TB Green® Premix Ex Taq™ II (TaKaRa, Code No. RR820A) according to manufacturer's instructions, respectively. The RT-qPCR conditions were as follows: initial denaturation at 95 °C for 30 s, followed by 40 cycles of 95 °C for 5 s, and annealing at 60 °C for 30 s. Elongation factor 1-alpha (*EF1α*) and tubulin 2 (*TUB2*) were used as internal reference genes^51^. The relative expression of the target gene was calculated by the 2^–△△Ct^ method^52^. The *Zfp1* gene expression were quantified by RT-qPCR using primer pair *Zfp1*-QF1/*Zfp1*-QR1, *EF1α*-QF/*EF1α*-QR, and *TUB2*-QF/*TUB2*-QR. The RT-qPCR assay was conducted with three independent biological and three technical replicates. All primers used in this study were listed in Table S1 and Fig. S1.

### Mycelial growth and conidiation assays

For mycelial growth, a 5-mm-diameter mycelial plug from a 3-day-old culture was placed on PDA and incubated at 28 °C for 5 days. For 5 days, the colony morphology was photographed, and the colony diameter was measured daily. For conidiation, a 5-mm-diameter mycelial plug from a 3-day-old culture was placed in PDB (200 mL) with shaking at 180 rpm and 28 °C. The conidial yield was calculated at 48, 72, and 96 h post-inoculation in PDB using a haemocytometer. Subsequently, conidia were filtered through two layers of lens paper and resuspended to a concentration of 1 × 10^6^ conidia·mL^-1^ in PDB. The conidial morphology was observed after 4 h of growth. The assays were performed with three biological replicates.

### Pathogenicity assays

*Polygonatum kingianum* plants collected from plantation in Kunming city of Yunnan province, China, and were permitted and identified by P. Ji from Institute of Medicinal Plant Cultivation. One drop of conidial suspension (1 × 10^6^ conidia·mL^-1^) was dripped onto the surface of each *P. kingianum* leaf and tuber^53,54^. Leaves/rhizomes inoculated with sterile water were used as the controls. Inoculated leaves and rhizomes were cultured on moist filter paper at 28 °C and 16-h light/8-h dark. Ten leaves and rhizomes were used for each treatment, with three biological replicates. Lesion diameters were measured at 5 days post-inoculation.

Moreover, the roots of one-year-old *P. kingianum* plants were dipped into conidial suspension (1 × 10^6^ conidia·mL^-1^) for 30 min. Plants whose roots were dipped in sterile water were used as the controls. Subsequently, the treatment and control plants were transplanted in pots filled with sterile soil and maintained in a growth chamber at 28 °C, 60% relative humidity, and 16-h light/8-h dark for 30 days. Disease index was calculated using the formula: [∑ (grade × number of plants corresponding grade) / (4 × total number of plants investigated)] × 100. Grade: 0 = healthy plants; 1 = yellowing of the lower leaves; 2 = yellowing of upper leaves; 3 = yellowing of most of the leaves; 4 = severe wilting or plant death^55^.

Experimental studies on plant samples, including the supply of plant material, comply with institutional, national and international guidelines and legislation.

### Response against stress

A 5-mm-diameter mycelial plug from a 3-day-old culture was placed on PDA supplemented with 1.8 M NaCl, 1.8 M KCl, 0.05% SDS, 0.1% CR, 0.08% H_2_O_2_, 0.25 μg·mL^-1^ tebuconazole, and 0.4 μg·mL^-1^ carbendazim. All the cultures were incubated in the dark at 28 °C. Subsequently, the colony diameters were measured daily until 5 days of growth. The inhibition ratio (%) was calculated as (C-N)/C × 100^56^, where C is the colony diameter of control and N is the colony diameter of the treatment. All treatments and the controls had three biological replicates, and 10 plates were applied for each replicate.

### Statistical analysis

The analysis of variance (ANOVA) was performed by IBM SPSS Statistics 26 (IBM Corporation, USA). Significance in all the comparisons among means with standard deviation was calculated by ANOVA with Duncan’s multiple comparison adjustment. Diagrams were made by GraphPad Prism 8 (GraphPad Prism Software Inc., San Diego, CA).

### Supplementary Information

Supplementary Information.

## Data Availability

The *Zfp1* nucleotide sequence was submitted to the NCBI database (https://www.ncbi.nlm.nih.gov/) with GeneBank accession number OR715798. The assembled genome sequences and raw reads of *F. oxysporum* PkF01 are accessible in the NCBI database under BioProject PRJNA835232 and in the Sequence Read Archive (SRA) under the accession number SRR19091468.

## Additional information

Correspondence and requests for materials should be addressed to L.Z.
