# Supplementary material for: Zinc finger transcription factor ZFP1 is associated with growth, conidiation, osmoregulation, and virulence in the Polygonatum kingianum pathogen Fusarium oxysporum
Source: Sci Rep. 2024 Jul 11;14:16061. doi: 10.1038/s41598-024-67040-7 (PMC11239662; doi:10.1038/s41598-024-67040-7)
Supplement: Supplementary file 1 — Supplementary Information. [file 41598_2024_67040_MOESM1_ESM.docx]

**Supplementary Information**

Table S1 List of primers used in this study.

Figure S1 Location of primers used in this study.

Figure S2 The original images of Figure 3A-D in this study.

**Table S1** List of primers used in this study

| Name | Sequence (5’-3’) | Product size (bp) |
| --- | --- | --- |
| *Zfp1*-UF | CAGCCGATACCGTCCAGAA | 678 |
| *Zfp1*-UR | TAGCCACGATTCGAAGCCGCTGCCGCAAGAGGTGGTTAT |  |
| *Zfp1*-DF | CGCATTGAATTGAAAAAGGAAGAGTATGACGCTGACGACCTTCTTCTT | 835 |
| *Zfp1*-DR | TGTCAAGTCGCTGCTCCAA |  |
| Hy-F | GCGGCTTCGAATCGTGGCTA | 800 |
| Hy-R | GTATTGACCGATTCCTTGCGGTCCGAA |  |
| Yg-F | GATGTAGGAGGGCGTGGATATGTCCT | 1112 |
| Yg-R | CATACTCTTCCTTTTTCAATTCAATGCG |  |
| *Zfp1*-IF | CTCAGCCAGCAACTCTCAG | 942 |
| *Zfp1*-IR | ATGTCAGCAGAAGCAGTAGC |  |
| *Zfp1*-UH-F | GGATGTGGTGGTGGTGGAT | 1100 |
| *Zfp1*-UH-R | CCGCTCGTCTGGCTAAGAT |  |
| *Zfp1*-DY-F | CCGTGGTTGGCTTGTATGG | 1216 |
| *Zfp1*- DY-R | TTGTGAGGATGAGCGTCTGT |  |
| *Zfp1*-CF | cccgggATGATGCCCCAGGCCGTCG | 1848 |
| *Zfp1*-CR | cccgggTTAGTCAGAGCGCTTGCGC |  |
| Neo-F | Atgattgaacaagatggattg | 880 |
| Neo-R | TCAGAAGAACTCGTCAAGAAG |  |
| *Zfp1*-QF1 | CGATGGCTTCAAGGAGGCTCTC | 136 |
| *Zfp1*-QR1 | GCTGGCGGTGTTGCTAAGAGA |  |
| *EF1α*-QF | GGTCAGGTCGGTGCTGGTTACG | 77 |
| *EF1α*-QR | TGGATCTCGGCGAACTTGCAGG |  |
| *TUB2*-QF | TTCTGCTGTCATGTCCGGTGT | 134 |
| *TUB2*-QR | TCAGAGGAGCAAAGCCAACCA |  |


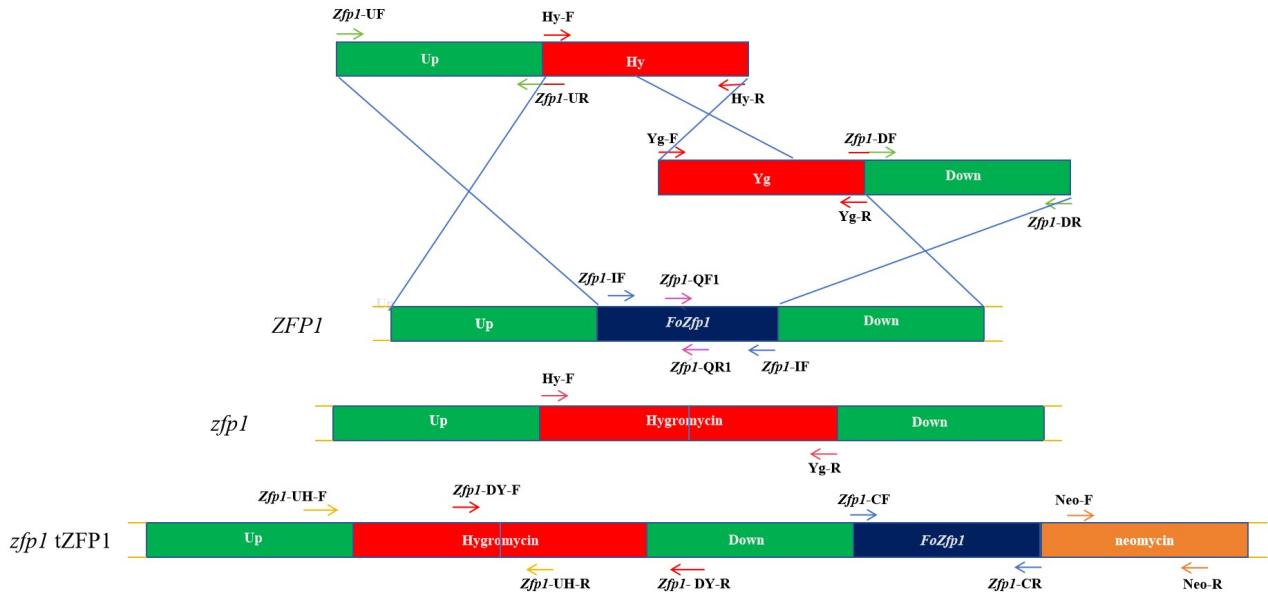


Figure S1 Location of primers used in this study.


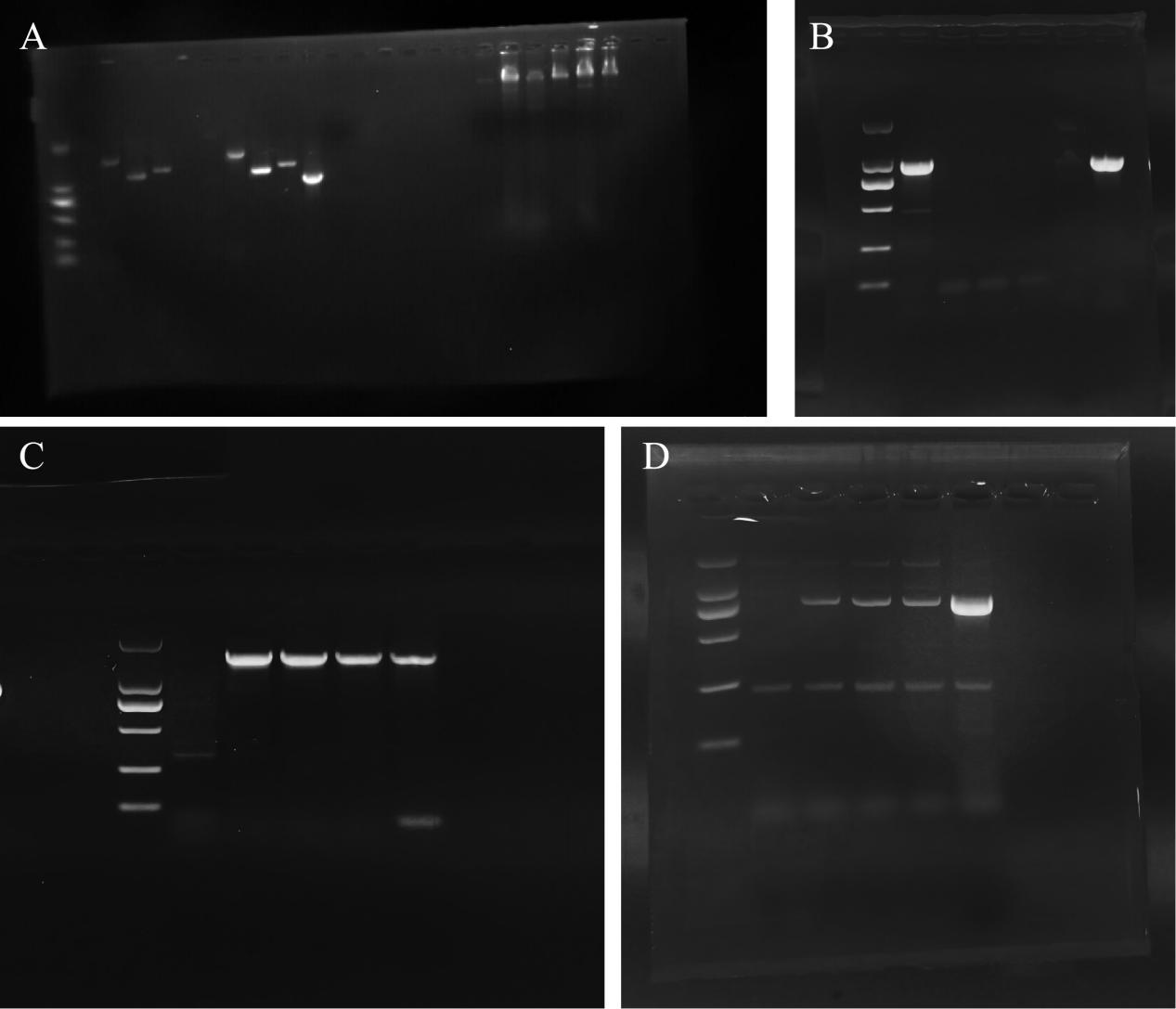


Figure S2 The original images of Figure 3A-D in this study.
